# EQ-5D-3L health state utility values in transfusion-dependent thalassemia patients in Malaysia: a cross-sectional assessment

**DOI:** 10.1186/s12955-020-01645-0

**Published:** 2021-01-07

**Authors:** Asrul Akmal Shafie, Irwinder Kaur Chhabra, Jacqueline Hui Yi Wong, Noor Syahireen Mohammed

**Affiliations:** 1 Discipline of Social and Administrative Pharmacy, School of Pharmaceutical Science, Universiti Sains Malaysia, 11800 Gelugor, Pulau Pinang Malaysia; 2 Institutional Planning and Strategic Centre, Universiti Sains Malaysia, 11800 Gelugor, Pulau Pinang Malaysia; 3 Pharmacy Department, Sabah Women and Children’s Hospital, Ministry of Health Malaysia, Karung Berkunci No.187, 88996 Kota Kinabalu, Sabah Malaysia; 4 Pharmacy Department, Hospital Kuala Lumpur, Ministry of Health Malaysia, Jalan Pahang, 50586 Kuala Lumpur, Wilayah Persekutuan Kuala Lumpur Malaysia; 5 Clinical Research Center, Hospital Sultanah Bahiyah, Ministry of Health Malaysia, KM 6, Jalan Langgar, 05460 Alor Setar, Kedah Darul Aman Malaysia

## Abstract

**Purpose:**

There is a gap of information describing the health state utility values (HSUVs) of transfusion-dependent thalassemia (TDT) patients in Malaysia. These values are useful in the assessment of health-related quality of life (HRQoL), economic evaluations and provide guidance to disease management decisions. The objective of this study was to estimate and derive HSUVs associated with the treatment and complications of TDT patients in Malaysia using the EQ-5D-3L instrument.

**Methods:**

A cross-sectional survey using the EQ-5D-3L instrument was conducted between May to September 2018 across various public hospitals in Malaysia. Using a multi-stage sampling, patients diagnosed with TDT and receiving iron chelating therapy were sampled. The findings on the EQ-5D-3L survey were converted into utility values using local tariff values. A two-part model was used to examine and derive the HSUVs associated with the treatment and complications of iron overload in TDT.

**Results:**

A total of 585 patients were surveyed. The unadjusted mean (SD) EQ-5D-3L utility value for TDT patients were 0.893 (0.167) while mean (SD) EQ VAS score was 81.22 (16.92). Patients who had more than two iron overload complications had a significant decline in HRQoL. Patients who were on oral monotherapy had a higher utility value of 0.9180 compared to other regimen combinations.

**Conclusion:**

Lower EQ-5D-3L utility values were associated with patients who developed iron overload complications and were on multiple iron chelating agents. Emphasizing compliance to iron chelating therapy to prevent the development of complications is crucial in the effort to preserve the HRQoL of TDT patients.

## Background

Thalassemia is the most prevalent hereditary hematologic disorder worldwide [1, 2]. Approximately 1.5% of the global population are estimated to be carriers of the β-thalassemia, with higher incidence in populations from the Mediterranean basin, Middle East, Indian subcontinent, Southeast Asia, Melanesia, and the Pacific Islands [3]. In Malaysia, there is an estimated carrier rate of 3.5–4.0% [4] and an estimated annual birth rate of 2.1/1000 [5]. As of August 2018, the Malaysian Thalassemia Registry reports that there are 7,984 thalassemia patients in the country and the numbers have increased since 2009 [6, 7]. Transfusion-dependent thalassemia (TDT) is an inherited blood disease characterized by the absence or decreased synthesis of one or more globin chains in the haemoglobin molecule, leading to chronic anaemia. Treatment for this condition involves a combination of blood transfusion to correct the anaemia, followed by iron chelating therapy to remove the excess iron resulting from the transfusion [8]. The thalassemia’s are likely to remain a global health problem in the future. There has been major efforts to better prevention and management of the disease [9].

The diagnosis of TDT has shown to affect a patient’s health-related quality of life (HRQoL) [10] and should be considered as an important index when evaluating treatment outcomes as it can be used to inform patient management and policy decisions [11]. Several studies have been conducted in Malaysia to assess the HRQoL of TDT patients between 2006 to 2013. Most of these studies utilized instruments such as the PedsQL 4.0 Generic Core Scales (GCS) [12–14] and Medical Outcomes Study Short Form 36-item (MOS SF-36) [15]. They showed that the diagnosis of TDT negatively impacts the HRQoL of patients. Patients undergo physical changes such as the development of thalassemia facies and stunted growth. The recurrent blood transfusion increases their risk of developing iron overload complications such as cardiac complications and diabetes, adding on to the disease burden [8]. The long-term use of medications, frequent hospital visits and fatigue brought about by the chronic anaemia disrupts their ability to function and can cause emotional distress [16]. However, these studies have been limited to the paediatric and adolescent TDT population. Apart from that, the HRQoL instruments used were non-preference-based instruments. Although these instruments quantify a health state in terms of functioning and symptoms, its scores are not weighted neither is there ‘value’ attached to it, making it difficult to use the outcomes in economic evaluations.

Value is measured in terms of ‘preference’ for a health state [17]. Health state utility values (HSUVs) represents an individual’s preference for being in a health state and can be measured indirectly using preference-based health-related quality of life instruments such as the EQ-5D. These utility values allow for meaningful comparison across different diseases and they are an important component of economic evaluations. The EQ-5D instrument was developed by the EuroQol Group and it consists of a descriptive system and a visual analogue scale (EQ VAS) [18]. The descriptive system has five dimensions (mobility, self-care, usual activities, pain/discomfort, anxiety/depression), each with either a 3-level response level (EQ-5D-3L) or a 5-level response level (EQ-5D-5L). A child-friendly version (EQ-5D-Y) has also been developed with a simpler language, suitable for use in children and adolescents. The responses on the descriptive system is used to form a unique health state which is subsequently converted into a single index value or a health state utility value (HSUV). The index value ranges from a score of 1 (referring to full health) and 0 (referring to a state as bad as being dead) or it may also take on negative values, representing health states considered to be worse than dead. These values are obtained from a standardized valuation exercise obtained from the general population in a country. This ensures that the values represent the societal perspective [18]. The 3L version of the instrument generates 243 possible health states whereas the 5L version generates 3125 possible health states. In Malaysia, both the 3L [19] and 5L [20] value sets are available, whereas the EQ-5D-Y value set is not yet available. In the second part of the instrument, the EQ VAS, respondents would indicate how they felt about their health on the day the survey was being performed. The EQ VAS is presented on a vertical health thermometer with 0 being the worst health imaginable, and 100 being the best health imaginable [18].

As of date, there is no study that describes the HSUV for the transfusion-dependent thalassemia population in the country. Thus, the objective of this study was to survey the HSUVs and health profiles of both paediatric and adult transfusion-dependent thalassemia (TDT) patients in Malaysia using the EQ-5D-3L instrument. The availability of a validated Malay version of the questionnaire and a country-specific general population tariff in Malaysia makes the use of the EQ-5D-3L instrument ideal. In addition to that, this study aims to derive HSUVs based on the iron chelating agent regimen used and the various iron overload complications associated with the condition. These HSUVs would be key drivers in cost effectiveness analyses as estimates of quality-adjusted life years (QALYs) are obtained by multiplying these utility values with the time spent in the health state.

## Methods

### Study design and participant recruitment

This cross-sectional study was conducted between May to September 2018 in Malaysia. Participants were selected using multi-stage sampling. In the first stage, Malaysia was divided into five regions based on its geographical location. In the second stage, a non-probability sampling was used to sample patients from each region. Tertiary referral hospitals which provide dedicated care for thalassemia patients were identified in each region and patients were recruited from these centres.

The sample size was determined using the population prevalence formula. Based on a population 7,984 [6], 5% precision rate, 95% confidence level and an assumption [21] of 50% disease prevalence, an estimated sample of at least 367 should be recruited. Recruitment criteria includes patients aged three years and above, a diagnosis of transfusion-dependent thalassemia, has received treatment of iron chelating therapy for at least six months and a proficiency in English or the Malay language to complete the surveys. Patients who defaulted treatment or regular follow up for at least a year, have impaired cognitive function, a diagnosis of non-transfusion dependent thalassemia or other hemoglobinopathies were excluded from this study.

### Data collection

One month prior to data collection, a nationwide training of interviewers was conducted to ensure consistency of data collection. Interviewers were given a set of forms consisting of a patient information sheet, informed consent forms for participation, parental consent form, age appropriate assent forms, EQ-5D-3L instrument and data collection form capturing patient’s sociodemographic data, and medical history. Both the Malay and English language version of the EQ-5D-3L instruments were used in this study. The instrument was previously validated in Malaysia [22] and a local value set is available [19]. Study coordinators visited study sites to randomly validate the data collection forms with the medical records to ensure accuracy of the collected data before the end of the data collection period.

Patients were screened and selected by the trained interviewers when they came in for routine follow up based on the inclusion and exclusion criteria. Prior to the face-to-face interview, both the patients and parents (if proxy-reported) were briefed about the objective of the study and assured that information collected would remain confidential. Informed consents and the child’s assent were obtained upon agreement to participate. Parents or caregivers were requested to answer the questionnaires on behalf of children aged between 3 and 12 years of age, as young children may not have the ability to decipher and respond to the questions. Adolescents above 12 years old were given the choice to self-report their own quality of life or if they could not, a parent proxy-report was done. Patients aged 18 years and above were expected to self-report their quality of life.

This study was registered with the National Medical Research Register (NMRR) of Malaysia (NMRR-17-2614-38966) and was approved by the Medical Research and Ethics Committee (MREC).

### Statistical analysis

Statistical analysis was conducted using STATA version 14 [23]. Descriptive statistical analysis including sum, percentage, mean and standard deviation was used to describe the sociodemographic, clinical factors, EQ-5D-3L domain responses, index and EQ VAS of the patients. The frequency and percentage of health profiles formed from the domain responses of the sample were also summarized. The EQ-5D-3L health profiles were then scored using the Malaysian EQ-5D-3L tariff set derived from a time-trade off multiplicative model [19, 24]. The distribution of EQ-5D-3L responses were summarized based on the population (pediatric or adult) and the source of the survey (self-reported or proxy-reported). Statistical significance of any reported problems between these groups would be examined using a Chi-square test. To summarize the responses of the EQ-5D-3L domains, the response was aggregated into ‘no reported problems’ and ‘reported problems’ by combining the response of ‘some problems’ and ‘extreme problems’. Statistical significance of the responses between the various demographic factors of patients would be examined using a Chi-square test.

The data normality of the EQ-5D-3L index and the EQ VAS was determined by observing the histogram and conducting a Shapiro–Wilk test. Depending on the normality data outcomes, statistical significance of differences between unadjusted means of the utility values and EQVAS scores would be tested (unpaired t-test or ANOVA if parametric and Mann–Whitney U test or Kruskal–Wallis test if non parametric). In addition to that, the calculation of effect sizes which measures the magnitude of the difference between the groups were performed. Cohen’s d measures the effect size by converting the difference between the means of two groups into standard deviation units (mean group 1 − means group 2/standard deviation). Values of (0.2–0.5), (0.5–0.8) and (> 0.8) corresponds to small, moderate and large differences in HRQoL [25].

The utility values are expected to be skewed, whereby many patients would report perfect health, as illustrated and discussed in previous studies [26, 27]. In this study, a two-part model was used to examine the association between the various groups of transfusion-dependent thalassemia patients and the disutility score (i.e. 1-EQ-5D-3L utility value) [27, 28]. The two-part model, which was estimated using STATAs *twopm* command, consists of two parts—a logistic regression and a generalized linear model [29]. The logistic regression first models the probability of disutility. The second part of the model utilized a generalized linear model with a gamma distribution and a log link function, as it showed the best fit based on comparisons of Akaike Information Criteria from various models. The analyses were adjusted for sociodemographic factors (age, gender, ethnicity, education level), number of iron overload complications, the specific coexisting complications, and the iron chelating therapy regimen. The analysis was conducted using the full sample (n = 585) and a restricted sample (n = 429). In the restricted sample, patients who reported perfect health but had iron overload complications were excluded from the analysis. Marginal effects (decrements of utility scores) based on the change in the demographic and treatment factors were estimated using STATAs margins command using the full sample.

Results were considered statistically significant for *p* < 0.05 in all the analysis.

## Results

The sociodemographic and clinical characteristics of the 585 patients sampled are described in Table 1. There were more females (55.7%) than male (44.3%) patients. The mean (SD) age of the sample was 17.2 (5.4) years, with an age range between 3 and 60 years old. Majority of the samples were of the Malay ethnicity. Since young children were also recruited for this study, the source of the HRQoL may have been self-reported (56.8%) or proxy-reported (43.2%). In both the self-reported and proxy reported groups, majority of the patients had received a primary or secondary education with 71.7% and 62.5% respectively. The mean (SD) age of initiating blood transfusion was 5.4 (7.9) years old while the mean (SD) number of transfusion years was 11.3 (8.9). A total of 56.6% of patients had no iron overload complications while the remaining 43.4% of patients reported the presence of at least one complication. Out of those who reported complications, 48.8% reported complications related to the liver. Majority of the patients were on monotherapy treatment (66.0%). The oral route of administration (56.6%) was the most common route of administration. Only 13.0% of the sampled population had any history of a serious adverse event with iron chelating therapy.

**Table 1:** Patients sociodemographic and clinical characteristics

Characteristics	Total (*N* = 585)
Age in years, mean (SD)	17.2 (5.4)
Age at first transfusion in years, mean (SD)	5.4 (7.9)
Number of years receiving blood transfusion, mean (SD)	11.3 (8.9)
Source of survey (%)	
Self-reported	332 (56.8)
Proxy-reported	253 (43.2)
Gender (%)	
Male	259 (44.3)
Female	326 (55.7)
Ethnicity (%)	
Malay	403 (68.9)
Chinese	94 (16.1)
Kadazan-Dusun	58 (9.9)
Others	30 (5.1)
Education level of proxy’s who completed proxy-report, n = 251 (%)	
No formal education	10 (4.0)
Primary or secondary education	180 (71.7)
Tertiary education	61 (24.3)
Education level of patients who completed self-report, n = 331 (%)	
No formal education	3 (0.9)
Primary or secondary education	207 (62.5)
Tertiary education	121 (36.6)
Presence of iron overload (IOL) complication (%)	
No complication	331 (56.6)
One complication	164 (28.0)
Two complications	66 (11.3)
Three or more complications	24 (4.1)
Cardiac disease	39 (15.3*)
Diabetes	20 (7.9*)
Hypothyroid	25 (9.8*)
Hypogonadism	90 (35.4*)
Hypoparathyroidism	52 (20.5*)
Liver disease	124 (48.8*)
Iron chelation therapy (%)	
Desferrioxamine (subcutaneous (SC) drug)	68 (11.6)
Deferasirox (oral (PO) drug)	245 (41.9)
Deferiprone (oral (PO) drug)	73 (12.5)
Desferrioxamine + Deferiprone (SC + PO drug)	154 (26.3)
Desferrioxamine + Deferasirox (SC + PO drug)	32 (5.8)
Deferiprone + Deferasirox (both oral drugs)	13 (2.2)
Number of iron chelating agents (%)	
Monotherapy	386 (66.0)
Dual therapy	199 (34.0)
Route of iron chelating administration (%)	
Subcutaneous	68 (11.6)
Oral	331 (56.6)
Subcutaneous + Oral	186 (31.8)
History of serious adverse event with iron chelation therapy (%)	
Yes	76 (13.0)
No	509 (87.0)

N, number; SD, standard deviation; IOL, iron overload complication; SC, subcutaneous; PO, oral

^*^Percentage calculated based on the total number of people who has complication

**Fig. 1:**
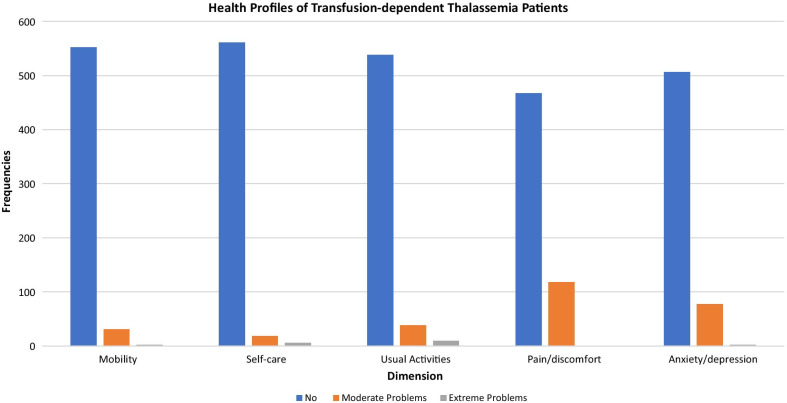
Health profiles of transfusion-dependent thalassemia patients

Out of the 243 possible health profiles with the EQ-5D-3L, 32 health profiles were reported, with 67.35% of patients reporting a health state of 11,111, followed by 10.09% reporting a health state of 11,121 (Additional file 1: Table 1). Figure 1 summarizes the distribution of health profiles for the sampled population. The pain/discomfort (20.2%) and the anxiety/depression (13.5%) domain had a higher percentage of reported problems compared to the other domains. Table 2 summarizes the distribution of EQ-5D-3L responses based on the population and source of survey. Based on the paediatric and adult population, the difference in score was significant on the mobility and self-care domains, with the pediatric population reporting more problems on the self-care domain while the adult population reported more problems on the mobility domain. Based on the source of the survey, there was a significant difference in the mobility, self-care and anxiety/depression domain. Respondents who self-reported reported more problems on the mobility and anxiety/depression domain whereas proxy-reported respondents reported more problems on the self-care domain.

**Table 2:** Distribution of EQ-5D-3L responses by population and source of survey

Dimension	By population category			By source of survey		
	Pediatric (n = 364)	Adult (n = 221)	*p* value	Self-report (n = 332)	Proxy-report (n = 253)	*p* value
Mobility						
Level 1	351 (96.4)	201 (91.0)		306 (92.2)	246 (97.2)	
Level 2	11 (3.0)	20 (9.1)		25 (7.5)	6 (2.4)	
Level 3	2 (0.6)	0 (0)		1 (0.3)	1 (0.4)	
Any problem in mobility (%)	13 (3.6)	20 (9.1)	0.005*	26 (7.8)	7 (2.8)	0.009*
Self-care						
Level 1	344 (94.5)	217 (98.2)		325 (97.9)	236 (93.3)	
Level 2	14 (3.9)	4 (1.8)		7 (2.1)	11 (4.4)	
Level 3	6 (1.7)	0 (0)		0 (0)	6 (2.4)	
Any problem in self-care (%)	20 (5.5)	4 (1.8)	0.029*	7 (2.1)	17 (6.7)	0.005*
Usual activities						
Level 1	338 (92.9)	200 (90.5)		306 (92.2)	232 (91.7)	
Level 2	20 (5.5)	18 (8.1)		23 (6.9)	15 (5.9)	
Level 3	6 (1.7)	3 (1.4)		3 (0.9)	6 (2.4)	
Any problem in usual activity (%)	26 (7.1)	21 (9.5)	0.309	26 (7.8)	21 (8.3)	0.836
Pain/discomfort						
Level 1	297 (81.6)	170 (76.9)		260 (78.3)	207 (81.8)	
Level 2	67 (18.4)	51 (23.1)		72 (21.7)	46 (18.2)	
Level 3	0 (0)	0 (0)		0 (0)	0 (0)	
Any problem in pain/discomfort (%)	67 (18.4)	51 (23.1)	0.172	72 (21.7)	46 (18.2)	0.295
Anxiety/depression						
Level 1	321 (88.2)	185 (83.7)		278 (83.7)	228 (90.1)	
Level 2	41 (11.3)	36 (16.3)		53 (16.0)	24 (9.5)	
Level 3	2 (0.6)	0 (0)		1 (0.3)	1 (0.4)	
Any problem in anxiety/depression (%)	43 (11.8)	36 (16.3)	0.125	54 (16.3)	25 (9.9)	0.025*

Values of scores are presented as n (%), number (percentage)

*p* value using Chi-square test

*indicates significance at *p* value < 0.05

A summary of the domain responses by sociodemographic and clinical factors based on aggregates of “no problem reported” and “problem reported” are shown in Additional file 1: Table 2. Using a Chi-square test, the aggregated domain responses were tested for statistical significance. Factors taken into consideration were gender, the presence of iron overload complications, the route and number of iron chelating (ICT) agents and the history of serious adverse events (SAE) with ICT use. On the mobility domain, all 5 factors were statistically significant whereas none of these factors were significant in the self-care domain. On the usual activity domain, the presence of iron overload complications, route, the number of iron chelating agents used and history of SAE were significant. On the pain/discomfort domain, only the presence of iron overload complications was significant. On the anxiety/depression domain, gender, presence of iron overload complications and the number of iron chelating agents used was significant.

**Fig. 2:**
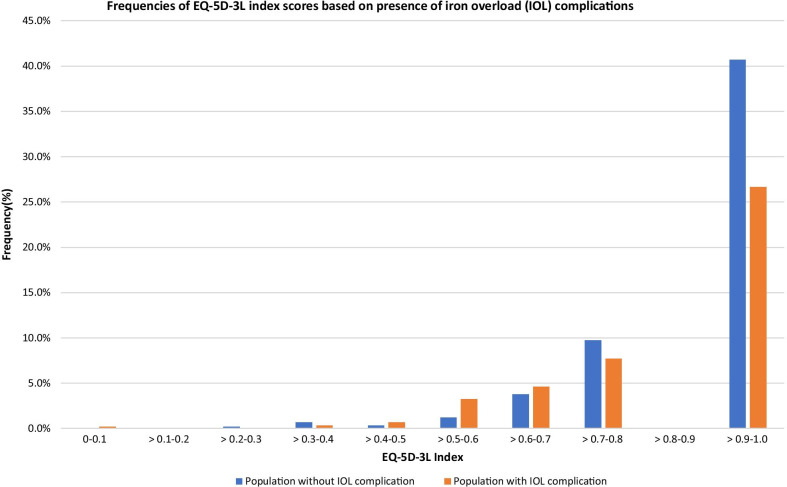
Frequencies of EQ-5D-3L utility values based on the presence of iron overload complications

Based on the number of iron overload complications, Fig. 2 illustrates the distribution of EQ-5D-3L utility values. Populations without iron overload complications tend to have a higher index score compared to populations with an IOL complication. The range of utility scores in the sample was limited between 0.4454 and 1.000.

The unadjusted mean (SD) EQ-5D-3L utility value for the entire sample was 0.893 (0.167) while the mean (SD) EQ VAS score was 81.22 (16.92), as summarized in Table 3. Based on the EQ-5D-3L utility values, statistical significance from Mann–Whitney and Kruskal–Wallis test found the variables of gender (effect size = 0.202), the presence of iron overload (effect size = 0.257), route of iron chelating therapy (effect size = 0.215 for oral and 0.06 for combination therapy compared to subcutaneous) and the number of iron chelating agents used (effect size = 0.274) to be statistically significant. However, the effect sizes of these variables were only small, with a coefficient that ranged less than 0.5, with the number of chelating agents used having the largest effect size.

**Table 3:** Summary of EQ-5D-3L mean utility values (unadjusted) and EQ VAS

Factors	EQ-5D-3L utility values				EQ VAS			
	Mean (SD)	Median (IQR)	*p* value^*¶*^	Effect size	Mean (SD)	Median (IQR)	*p* value^*¶*^	Effect size
Total sample	0.893 (0.167)	1.000 (0.269)	–	–	81.22 (16.92)	85.00 (25.00)	–	–
Source of survey								
Self-reported	0.887 (0.165)	1.000 (0.269)	0.219	0.080	78.08 (18.19)	80.00 (20.00)	< 0.001*	0.439
Proxy-reported	0.900 (0.169)	1.000 (0.255)			85.36 (14.10)	90.00 (15.00)		
Gender								
Male	0.911 (0.157)	1.000 (0.244)	0.011*	0.202	82.29 (15.53)	85.00 (20.00)	0.450	0.114
Female	0.878 (0.173)	1.000 (0.269)			80.37 (17.93)	85.00 (25.00)		
Category								
Child (≤ 18 years old)	0.898 (0.163)	1.000 (0.255)	0.079	0.371	84.97 (15.16)	90.00 (15.00)	< 0.001*	0.611
Adult	0.884 (0.172)	1.000 (0.269)			75.04 (17.86)	80.00 (25.00)		
Presence of iron overload complication								
Absent	0.911 (0.153)	1.000 (0.244)	0.002*	0.257	83.31 (16.61)	85.00 (15.00)	< 0.001*	0.288
Present	0.869 (0.180)	1.000 (0.269)			78.49 (16.70)	80.00 (20.00)		
Route of iron chelating therapy								
Subcutaneous (SC) only	0.877 (0.187)	1.000 (0.269)	0.016^¥,^*	Reference Group	78.45 (15.68)	80.00 (20.00)	< 0.001^¥,^*	Reference Group
Oral (PO) only	0.911 (0.151)	1.000 (0.244)		0.215	83.82 (15.41)	90.00 (15.00)		0.347
SC + PO	0.866 (0.181)	1.000 (0.269)		0.060	77.60 (19.06)	80.00 (20.00)		0.047
Number of iron chelating agents								
Monotherapy	0.908 (0.153)	1.000 (0.255)	0.005*	0.274	82.95 (15.52)	87.50 (20.00)	< 0.001*	0.299
Dual therapy	0.863 (0.186)	1.000 (0.269)			77.90 (18.95)	80.00 (20.00)		
History of serious adverse event with iron chelating therapy								
Yes	0.881 (0.181)	1.000 (0.269)	0.585	0.078	78.16 (17.52)	85.00 (20.00)	0.068	0.208
No	0.894 (0.164)	1.000 (0.269)			81.67 (16.80)	80.00 (20.00)		

Values of scores are presented as Mean (SD; standard deviation); Median (IQR; interquartile range) N, number; SC, subcutaneous; PO, oral; VAS, Visual Analogue Scale

^¶^*p *value using Mann Whitney U Test

^¥^*p* value using Kruskal Wallis Test

^*^indicates significance at *p* value < 0.05, Effect Size (Cohen’s d Interpreted as *d* = 0.2–0.5 (Small), *d* = 0.5–0.8 (Moderate) and *d* > 0.8 (Large)

Based on the EQ VAS, the Mann–Whitney and Kruskal–Wallis test found the source of survey (effect size = 0.439), category of child or adult (effect size = 0.611), the presence of iron overload (effect size = 0.288), route of iron chelating therapy (effect size = 0.347 for oral and 0.047 for combination therapy compared to subcutaneous) and the number of iron chelating agents used (effect size = 0.299) to be statistically significant. The effect size of the child or adult category was the largest with a value of 0.611.

Table 4 presents the HSUVs for the different types of iron chelating agents and specific iron overload complications which were generated using a two-part model, controlling for gender and the presence of iron overload complications in the model. The analysis was conducted using the full sample (n = 585) and a restricted sample (n = 429). In the restricted sample, patients who reported perfect health but had iron overload complications were excluded from the analysis. We hypothesized that patients who had iron overload complications would not be able to achieve perfect health and hence wanted to examine what the HSUVs would be without that cohort. Using the full sample, patients who had diabetes had the lowest utility value of 0.815 compared to other iron overload complications. However, when the restricted sample was used, patients with hypothyroid had the lowest utility value of 0.838. In both sample analysis, patients who were on oral monotherapy had a higher utility value compared to other routes of administration. Based on the full sample, the health state utility values using a subcutaneous iron chelator (0.893) resulted in a utility decrement of 2.7% compared to when an oral iron chelator (0.918) is used, whereas patients in the restricted sample had a utility decrement of 17.9%. In addition to the route of administration, the number of iron chelators used can affect the HRQoL of patients, whereby patients who had more than one iron chelator had lower utility values compared to those who had monotherapy in both the samples.

**Table 4:** Health state utility values derived from the two-part model

Health state	Full sample (n = 585)			Restricted sample (n = 429)		
	Utility value	95% CI		Utility value	95% CI	
TDT with non-specific iron overload complication	0.852	0.811	0.893	0.905	0.880	0.929
TDT with cardiac complication	0.820	0.742	0.897	0.846	0.805	0.887
TDT with diabetes	0.815	0.711	0.919	0.839	0.788	0.890
TDT with hypothyroid	0.853	0.761	0.945	0.838	0.787	0.889
TDT with hypogonadism	0.841	0.785	0.896	0.860	0.827	0.893
TDT with hypoparathyroidism	0.894	0.832	0.955	0.856	0.815	0.897
TDT with liver iron overload	0.826	0.778	0.875	0.929	0.907	0.951
TDT with desferrioxamine (DFO)	0.893	0.880	0.906	0.779	0.707	0.852
TDT with deferasirox (DFX)	0.915	0.856	0.974	0.854	0.837	0.870
TDT with deferiprone (DFP)	0.940	0.875	1.005	0.778	0.698	0.859
TDT with DFO + DFP	0.903	0.841	0.964	0.749	0.691	0.808
TDT with DFO + DFX	0.862	0.229	0.953	0.758	0.662	0.854
TDT with DFX + DFP	0.814	0.653	0.975	0.754	0.591	0.917
TDT on subcutaneous (SC) therapy	0.893	0.879	0.906	0.779	0.706	0.852
TDT on oral (PO) therapy	0.918	0.860	0.976	0.949	0.902	0.997
TDT on SC + PO therapy	0.895	0.834	0.957	0.890	0.837	0.942

CI, confidence interval; TDT, transfusion-dependent thalassemia; DFO, desferrioxamine; DFX, deferasirox; DFP, deferiprone; SC, subcutaneous; PO, oral

The marginal effects of the various sociodemographic and clinical factors on the disutility score were analysed using STATA’s two-part model margins command on the full sample (Additional file 1: Table 3). Gender, length of transfusion, presence of iron overload complications and number of iron chelating agents used were found to be significant predictors for the disutility score. When comparing the EQ-5D-3L utility values of patients with complications to patients without complications in the two-part model, the HRQoL declining effect becomes significant when there are at least two coexisting complications. Patients who used dual iron chelating agents had lower utility scores by 0.15 points when compared to those who were on monotherapy.

## Discussion

As of date, there are no studies that has surveyed the health state utility values (HSUV) of transfusion-dependent thalassemia (TDT) patients in Malaysia and this study aims to fill that gap. The availability of these values would aid policy makers in the decision-making process related to disease management for these group of patients, especially since TDT is a chronic and expensive disease.

A ceiling effect was seen in the sample with 67.35% of patients reporting perfect health and this may be attributed to the EQ-5D-3L instrument used. Although the EQ-5D-5L instrument has been shown to be superior compared to the EQ-5D-3L [30], the EQ-5D-3L instrument was chosen because of studies which support the validity of its use in children [31–33] in the absence of the EQ-5D-Y value sets, since both the pediatric and adult population was sampled. In addition to that, a 3-level response would be simpler to administer compared to a 5-level response.

This study found differences between the domain responses of pediatric and adult patients. The lower number of reported problems on the self-care domain of older patients may be explained by the independence and knowledge that the patient gains over the years when coping with the condition [13, 34]. Pediatric patients also tend to be more dependent on their caregivers for daily tasks, hence explaining the higher number of reported problems on the self-care domain. As patients grew older, the burden of treatment may increase with higher volume of blood required, onset of complications and the need for higher dosages of iron chelating therapy, possibly contributing to the higher number of reported problems on the mobility and anxiety/depression domain, although the difference was only significant in the mobility domain. Respondents who self-reported reported higher number of problems in the mobility and anxiety/depression domain. This is expected as they would be able to perceive better a limitation in their daily routine. Respondents who proxy-reported reported a higher number of problems on the self-care domain, as caregivers are the one who would be helping patients in their self-care routine, and would be less able to perceive the other domains which may not be observed physically such as pain/discomfort and anxiety/depression.

The mean (SD) HSUV of 0.893 (0.167) in this study was slightly higher than the HSUVs surveyed among TDT patients using iron chelating therapy in Iran. The study, which utilized US and Iran’s time trade-off value set, had a HSUV range of 0.81 to 0.86 [35]. A similar trend was also observed on the EQ VAS scale, where our patients had a mean (SD) score of 81.22 (19.92) compared to a score of 72.9 (1.1) in Iran [35]. The utility value range between 0.8 to 0.9 had no reported frequencies as the plausible range on the Malaysian EQ-5D-3L tariff set had a gap between the health state 11,111 (utility value = 1.00) and 11,112 (utility value = 0.756) [19].

The distribution of utility values sampled were skewed, with many patients reporting perfect health and this was also evident in previous studies [26, 27]. The skewed distribution violates ordinary least squares (OLS) regression assumptions. Hence, a two-part model (TPM) was used to derive the HSUVs, controlling for age, the type of iron overload and type of iron chelator. In the two-part model, the disutility score (i.e. 1-EQ-5D-3L utility value) [27, 28] was used to predict the HRQoL scores based on the variables. The two-part model is useful for models with mixed discrete continuous outcomes. In this study, the first part of the two-part model predicts the probability of obtaining a disutility score of 0(perfect health), followed by the second part of the model that predicts the disutility score using a generalized linear model (GLM) [29]. The GLM model was chosen as it allows the outcome variable to be a link function of the linear index of the covariates instead of the outcome variable simply being a linear function of the covariates. This also avoids the problem of retransformation inherent in models that transformed the outcome variable to meet OLS assumptions [36].

Based on the full and restricted sample, the health state utility values using a subcutaneous iron chelator resulted in a utility decrement of 2.7% and 17.9% compared to when an oral iron chelator is used. The study conducted in Iran showed a decrement of 6.9% when using a subcutaneous iron chelator or combination therapy compared to an oral chelator or a monotherapy [35]. Although these findings of decrement are consistent with previous studies, the utility decrement in this study and the study conducted in Iran is much smaller compared to a time trade-off study conducted in Australia [37] and the United Kingdom [38] that investigated the utility associated with the use of oral and subcutaneous iron chelating therapy. In both studies, a decrement of 28.2% and 21.4% respectively occurred when using a subcutaneous ICT compared to the oral ICT. However, it should be noted that these two studies utilized a direct elicitation valuation method with respondents from the community, while the current study and the study in Iran utilized a generic preference-based instrument with TDT patient. This highlights the effect of different valuation methods and respondents in eliciting HSUVs.

Amongst the various iron overload complications, cardiac and diabetes complications result in the highest disutility of 8.2% and 8.4% (full sample) or 6.5% and 7.3%(restricted sample) respectively compared to respondents without complications. These values were higher compared to the study conducted in Iran which had a disutility of 3.6% (cardiac) and 6.0% (diabetes) when compared to patients who did not have the complication [35]. A cost-utility analysis conducted in the United States used the assumption that TDT patients with cardiac disease would have a 15% decrement in utility [39] based on TTO values for heart failure as reported in a longitudinal cohort study of health status and HRQoL [40]. These findings imply that the prevention of iron overload complications is crucial in preserving the HRQoL of patients. Based on the marginal effects, as with other chronic conditions [41], this study showed that the presence of coexisting comorbidities in TDT is associated with a lower HRQoL. The decrease in HRQoL becomes statistically significant when the number of complications exceeds two. Compliance to iron chelation therapy is essential in the prevention of the development of iron overload complications. Apart from the increased risk of morbidity and mortality, poor compliance to iron chelating therapy has also shown to increase the cost of treating the disease [42]. The choice of iron chelators has been highlighted as a determinant of HRQoL in TDT patients [43–45] and the findings of this study further emphasizes the preference for oral iron chelation.

This study has a few limitations. First, it was not compared to a healthy general population, hence making it difficult to truly estimate the impact of the disease on the HRQoL. In addition, due to time constraints and the variation in documentation of medical records across centres, it was a challenge to obtain additional clinical parameters which could have been used to assess the severity of the disease such as the serum ferritin levels. As is common for quality of life data, the results were skewed in a way that higher scores were reported more than lower scores. A ceiling effect was also seen in our results (67.35% reported perfect health) indicating that a complete variation in health states were not fully captured. The use of EQ-5D-3L in children also presents methodological concerns such as the relevance of the domains, appropriateness of the instrument based on the cognitive ability and the concordance between self and proxy reports. Agreement between the self-report and proxy-report was also not examined as respondents were only surveyed once. Unfortunately, the absence of other suitable utility tariffs in the country, the evidence that support the validity of EQ-5D-3L use in children [31–33] and the simple administration of the EQ-5D-3L instrument made it attractive for use. Finally, based on a literature search, the minimum clinically importance difference (MCID) which represents the smallest amount of benefit that the patient can recognize and value has not been defined for TDT. This limits our ability to compare the actual impact of using different treatment regimens and route of administrations on the HRQoL and to assess if patients have significantly improved, declined or remained stable [46]. Further empirical work is required in this area.

## Conclusion

In this study, the HRQoL of TDT patients in Malaysia were surveyed using the EQ-5D-3L instrument and converted into utility values using a Malaysian specific tariff. The mean (SD) EQ-5D-3L utility value for TDT patients were 0.893 (0.167) while the mean (SD) EQ VAS score was 81.22 (16.92). The reduction of HRQoL becomes more prominent when there are at least two coexisting complications. These findings emphasize the importance of preventing the development of iron overload complications. However, the prevention of iron overload is heavily dependent on a patient’s compliance to their iron chelating therapy. The availability of these values would be useful for clinicians, researchers and policy makers in the decision-making process for disease management.

## Supplementary Information

**Additional file 1**. Frequencies of health profiles reported.

## Data Availability

The datasets used and/or analysed during the current study are available from the corresponding author on reasonable request.
