# Supplementary material for: EQ-5D-3L health state utility values in transfusion-dependent thalassemia patients in Malaysia: a cross-sectional assessment
Source: Health Qual Life Outcomes. 2021 Jan 7;19:10. doi: 10.1186/s12955-020-01645-0 (PMC7791838; doi:10.1186/s12955-020-01645-0)
Supplement: Supplementary file 1 — Additional file 1. Frequencies of health profiles reported. [file 12955_2020_1645_MOESM1_ESM.docx]

**Additional Files:**

**Additional Files Table 1: Frequencies of health profiles reported**

| **Health Profile** | **Frequency** | **Percent (%)** |
| --- | --- | --- |
| 11111 | 394 | 67.35 |
| 11112 | 29 | 4.96 |
| 11113 | 1 | 0.17 |
| 11121 | 59 | 10.09 |
| 11122 | 21 | 3.59 |
| 11211 | 10 | 1.71 |
| 11212 | 3 | 0.51 |
| 11221 | 7 | 1.2 |
| 11222 | 6 | 1.03 |
| 11311 | 1 | 0.17 |
| 11321 | 1 | 0.17 |
| 12111 | 4 | 0.68 |
| 12112 | 1 | 0.17 |
| 12122 | 4 | 0.68 |
| 12211 | 2 | 0.34 |
| 12221 | 1 | 0.17 |
| 12311 | 1 | 0.17 |
| 12312 | 1 | 0.17 |
| 13111 | 2 | 0.34 |
| 13211 | 1 | 0.17 |
| 13221 | 1 | 0.17 |
| 13311 | 2 | 0.34 |
| 21111 | 14 | 2.39 |
| 21121 | 3 | 0.51 |
| 21122 | 5 | 0.85 |
| 21221 | 1 | 0.17 |
| 21222 | 3 | 0.51 |
| 21322 | 2 | 0.34 |
| 22222 | 2 | 0.34 |
| 22223 | 1 | 0.17 |
| 31111 | 1 | 0.17 |
| 32321 | 1 | 0.17 |
| **Total** | 585 | 100 |

**Additional Files Table 2: Summary of dimension responses**

| **Demographics** | **Mobility n (%)** | | | **Self-care n (%)** | | | **Usual activity n (%)** | | | **Pain/discomfort n (%)** | | | **Anxiety/depression n (%)** | | |
| --- | --- | --- | --- | --- | --- | --- | --- | --- | --- | --- | --- | --- | --- | --- | --- |
|  | **No Problem** | **Reported Problem** | **p-value** | **No Problem** | **Reported Problem** | **p-value** | **No Problem** | **Reported Problem** | **p-value** | **No Problem** | **Reported Problem** | **p-value** | **No Problem** | **Reported Problem** | **p-value** |
| **Gender** |  |  | 0.017* |  |  | 0.564 |  |  | 0.39 |  |  | 0.087 |  |  | <0.001* |
| Male | 251(96.9) | 8(3.1) |  | 247(95.4) | 12(4.6) |  | 241(93.1) | 18(6.9) |  | 215(83.0) | 44(17.0) |  | 240(92.7) | 19(7.3) |  |
| Female | 301(92.4) | 25(7.6) |  | 314(96.3) | 12(3.7) |  | 297(91.1) | 29(8.9) |  | 252(77.4) | 74(22.6) |  | 266(81.7) | 60(18.4) |  |
| **Presence IOL** |  |  | 0.04* |  |  | 0.860 |  |  | 0.020* |  |  | 0.002* |  |  |  |
| Yes | 234(92.1) | 20(7.9) |  | 244(96.2) | 10(3.8) |  | 226(89.0) | 2(11.0) |  | 188(74.0) | 66(26.0) |  | 209(82.3) | 45(17.7) | 0.009* |
| No | 318(96.1) | 13(3.9) |  | 317(95.8) | 14(4.2) |  | 312(94.3) | 19(5.7) |  | 279(84.3) | 52(15.7) |  | 297(89.7) | 34(10.3) |  |
| **ICT Route** |  |  | 0.006* |  |  | 0.370 |  |  | 0.028* |  |  | 0.248 |  |  | 0.058 |
| SC | 63(92.7) | 5(7.3) |  | 67(98.5) | 1(1.5) |  | 63(92.7) | 5(7.3) |  | 51(75.0) | 17(25.0) |  | 57(83.8) | 11(16.2) |  |
| PO | 321(97.0) | 10(3.0) |  | 318(96.1) | 13(3.9) |  | 312(94.3) | 19(5.7) |  | 272(82.2) | 59(17.8) |  | 296(89.4) | 35(10.6) |  |
| SC + PO | 168(90.3) | 18(9.7) |  | 176(94.6) | 10(5.4) |  | 163(87.6) | 23(12.4) |  | 144(77.4) | 42(22.6) |  | 153(82.3) | 33(17.7) |  |
| **No of ICT Agents** |  |  | 0.001* |  |  | 0.212 |  |  | <0.001* |  |  | 0.136 |  |  | 0.020* |
| Monotherapy | 373(96.6) | 13(3.4) |  | 373(96.6) | 13(3.4) |  | 367(95.1) | 19(4.9) |  | 315(81.7) | 71(18.3) |  | 343(88.9) | 43(11.1) |  |
| Dual Therapy | 179(90.0) | 20(10.0) |  | 188(94.5) | 11(5.5) |  | 171(85.9) | 28(14.1) |  | 152(76.4) | 47(23.6) |  | 163(81.9) | 36(18.1) |  |
| **History of SAE** |  |  | 0.048* |  |  | 0.942 |  |  | 0.027* |  |  | 0.919 |  |  | 0.925 |
| Yes | 68(89.5) | 8(10.5) |  | 73(96.0) | 3(4.0) |  | 65(85.5) | 11(14.5) |  | 61(80.3) | 15(19.7) |  | 66(86.8) | 10(13.2) |  |
| No | 484(95.1) | 25(4.9) |  | 488(95.9) | 21(4.1) |  | 473(92.9) | 36(7.1) |  | 406(79.8) | 103(20.2) |  | 440(86.4) | 69(13.6) |  |

_Values of scores are presented as n(%), number(percentage);IOL, Iron Overload Complications; ICT, Iron Chelating Therapy; SC, subcutaneous; PO, oral; SAE, Severe Adverse Effects_  *_p-value using Chi-square test;* indicates significance at p-value < 0.05_*

**Additional Files Table 3: Marginal effects estimated with a two-part model for the association between sociodemographic and clinical factors and the EQ-5D-3L disutility score (i.e. 1 – EQ-5D-3L utility value)**

|  | **Disutility score** | **Standard Error** | **p-value** | **95% Lower** | **95% Upper** |
| --- | --- | --- | --- | --- | --- |
| **Age** | 0.108 | 0.001 | 0.197 | 0.094 | 0.123 |
| <10 years old as reference group | | | | | |
| 11-20 Years Old | 0.117 | 0.016 | 0.607 | 0.072 | 0.162 |
| 21-30 Years Old | 0.107 | 0.019 | 0.999 | 0.055 | 0.159 |
| 31-40 Years Old | 0.141 | 0.027 | 0.431 | 0.073 | 0.208 |
| 41-50 Years Old | 0.260 | 0.054 | 0.012* | 0.141 | 0.380 |
| >50 Years Old | 0.118 | 0.065 | 0.615 | -0.022 | 0.259 |
| **Gender** | | | | | |
| Female as reference group | | | | | |
| Male | 0.073 | 0.013 | 0.036* | 0.033 | 0.114 |
| **Age initiated transfusion** | 0.108 | 0.001 | 0.695 | 0.092 | 0.122 |
| **Years of transfusion** | 0.109 | 0.001 | 0.040* | 0.094 | 0.124 |
| **Presence of Iron Overload Complications** | | | | | |
| IOL Presence | 0.149 | 0.014 | 0.009* | 0.108 | 0.190 |
| **Number of coexisting iron overload complications** | | | | | |
| No complication as reference group | | | | | |
| 1 Complication | 0.129 | 0.015 | 0.312 | 0.086 | 0.173 |
| 2 Complications | 0.162 | 0.023 | 0.036* | 0.103 | 0.221 |
| 3 or more complications | 0.250 | 0.045 | 0.001* | 0.147 | 0.353 |
| **Number of Iron Chelating Agents** | | | | | |
| Monotherapy as reference group | | | | | |
| Dual Therapy | 0.152 | 0.015 | 0.004* | 0.109 | 0.196 |
| **Route of iron chelating therapy administration** | | | | | |
| Subcutaneous route as reference group | | | | | |
| Oral | 0.073 | 0.022 | 0.281 | 0.015 | 0.131 |
| Combination of oral & subcutaneous | 0.118 | 0.025 | 0.907 | 0.055 | 0.180 |
| **History of Serious Adverse Event (SAE) with Iron Chelating Therapy (ICT) use** | | | | | |
| Did not have a history of SAE with ICT use as reference group | | | | | |
| Experienced SAE with ICT use | 0.120 | 0.022 | 0.326 | 0.063 | 0.177 |

_IOL, Iron Overload Complications; ICT, Iron Chelating Therapy; SC, subcutaneous; PO, oral; SAE, Severe Adverse Effects_*_;* indicates significance at p-value < 0.05_*
